# Editorial: New emerging functions of transcription factors and RNA-binding proteins in the development of hematological malignancies

**DOI:** 10.3389/fonc.2023.1256461

**Published:** 2023-08-01

**Authors:** Tarik Möröy, Cyrus Khandanpour

**Affiliations:** ^1^ Institut de recherches cliniques de Montreal (IRCM), Montreal, QC, Canada; ^2^ Department de Microbiologie, Infectiologie et Immunologie, Université de Montréal, Montreal, QC, Canada; ^3^ Division of Experimental Medicine, McGill University, Montreal, QC, Canada; ^4^ Department of Hematology and Oncology, University of Lübeck and University Hospital Schleswig-Holstein Campus Lübeck, Lübeck, Germany

**Keywords:** transcription factors, RNA-binding proteins, leukemia, lymphoma, hematopoietic stem cells

Hematological malignancies such as acute or chronic leukemias and lymphomas belong to a group of cancers that affect the blood, bone marrow and peripheral lymphoid organs and continue to present significant challenges for diagnosis and treatment primarily because of their diversity and heterogeneity. Recent research has shed light on the roles of transcription factors (TFs) and RNA-binding proteins (RBPs), that both are intimately linked to the development and progression of hematological malignancies because they control the generation, stability and translation of mRNA. This editorial aims to summarize the findings and conclusions from a set of six articles available in the Frontiers Research Topic “*New Emerging Functions of Transcription Factors and RNA-Binding Proteins in the Development of Hematological Malignancies*” on Frontiers in Oncology.

The functionality and longevity of hematopoietic tissue from which leukemia and lymphoma can emerge is ensured by a tightly controlled balance between self-renewal, quiescence, and differentiation of hematopoietic stem cells (HSCs) into the many different blood lineages. Benyoucef et al. describe how Transcription Factors (TFs) regulate this by translating signals from extrinsic factors into specific gene expression programs. Any aberration or mutation that alters the function of TFs will affect HSCs and their ability to self-renew or differentiate and thus bear the risks to trigger transformation processes that lead to a malignancy. When TFs are mutated or activated in hematological malignancies, they are responsible for specific gene expression patterns that are different from those found in normal cells. These aberrations can be measured and, in many cases, initiate a leukemia or lymphoma or drive their progression. However, they are not only characteristic for certain types of leukemia, but they provide a typical, recurring signature, that can be used for diagnosis or to make a prognostic estimate.

A new example for TF action in hematological malignancies is given by (Zhang et al.). Their article reveals new actionable therapeutic targets such the protein “Fifth Ewing Variant” (FEV), which was found to be activated in 30% of primary samples from patients with an ongoing acute myeloid leukemia (AML) and in nearly all samples from patients with an AML relapse after therapy. FEV belongs to the ETS family of TFs and regulates the homing and expansion of AML cells by activating the expression of integrin α4. Of interest is the observation that when integrin α4 is blocked with the antibody natalizumab (NZM), migration and colony-forming abilities of leukemia blasts and leukemic-initiating cells (LICs) are reduced suggesting that the link between FEV and integrin α4 can be a therapeutic target for AML.

TFs bind to specific DNA sequences and interact with other regulatory proteins such as epigenetic regulators for example the enzyme Lysine specific demethylase 1 (LSD1), which catalyses the removal of methyl groups from Histone H3 lysine 4, and thereby enables chromatin compaction and transcriptional repression. Hartung et al. describe how TFs recruit LSD1 to chromatin to maintain a gene expression program that ensures the balance between HSC self-renewal, proliferation and differentiation into the myeloid lineage. A number of small molecule LSD1 inhibitors are currently clinically tested with the hope to drive AML blast cells to differentiate and to render them more vulnerable to chemotherapy. The review describes the effect of inhibitors on the action of TFs that either directly or indirectly interact with LSD1 such as GFI1, GFI1B, PU.1 and C/EBPα as well as TFs that are regulated downstream of LSD1 such as IRF8. The review also addresses the question how the combination of LSD1 inhibitors with other chemotherapeutic drugs could lead to better patient outcome.

While TFs regulate the abundance of mRNAs and other transcripts, RNA-binding proteins participate in the control of gene expression by regulating mRNA processing, -transport, -stability, and -translation. The article from Zhao et al. reviews the structural and functional characteristics of Poly(rC)-binding proteins (PCBPs) and hnRNP K. These proteins belong to a subfamily of RNA binding proteins known for their role in hematopoietic cell growth, differentiation, and tumorigenesis. PCBP1-4 and hnRNP K share the so-called K Homology (KH) domain first identified in the human hnRNP K and is an evolutionarily conserved sequence of 70 amino acids. The KH domain can bind to specific mRNA molecules and regulates their stability or their alternative splicing or affect their translation. Interestingly, PCBPs are involved in both myeloid acute and chronic leukemias and also in lymphoid malignancies affecting major oncogenic pathways involving the oncoprotein c-MYC, the tumor suppressor TP53 and regulators of cell cycle progression.

Another RNA binding protein, which has recently gained notoriety as a new player in hematological malignancies, is the RNA helicase DDX3X. Somatic loss of function mutations in the X chromosome-located *DDX3X* gene were found in B-cell lymphomas, particularly those with *c-MYC* translocations. Notably, these mutations were almost exclusively observed in males and not in females, and the male homologue *DDX3Y* (located on the Y chromosome) was never found to be mutated in any malignancy. Lacroix et al. summarize in their review new findings indicating that loss of function of DDX3X assists the initial process of B-cell lymphomagenesis, but the completion of lymphomagenesis requires DDX3 activity and in the absence of a function DDX3X leads to upregulation of DDX3Y. The findings suggested that DDX3Y could serve as a cancer cell-specific target for developing adjuvant therapies for male patients with DDX3X mutations in B-cell lymphomas.

The third review on RNA binding proteins focuses on myelodysplastic syndrome (MDS), a heterogeneous disorder of the blood cells. Specifically, Ma et al. investigated MDS patients with SF3B1 mutations, which are considered a favorable prognostic biomarker. The study reviewed 140 MDS patients from the Zhejiang province of China and found that RUNX1, EZH2, NF1, and KRAS/NRAS mutations significantly impacted overall survival (OS) in addition to the commonly co-mutated TET2, ASXL1, and DNMT3A genes. Based on these findings, the researchers developed a risk scoring model that outperformed the widely used International Prognostic Scoring System (IPSS-R). This personalized prediction model categorized patients into low-risk and high-risk groups, providing a more accurate assessment of their 3-year overall survival.

The research showcased in this Research Topic represents significant progress in unraveling the intricate roles of transcription factors and RNA-binding proteins in hematological malignancies. Advancements in gene-editing technologies, such as CRISPR-Cas9, and the discovery of small molecules that selectively inhibit TF and RBP interactions hold promise for ever better precision medicine approaches in hematological malignancies. It is clear that studying these proteins provides new insight that will pave the way for the development of innovative therapeutic strategies and hold great potential for improving the diagnosis, treatment, and management of patients with hematological malignancies.

## Author contributions

All authors listed have made a substantial, direct, and intellectual contribution to the work and approved it for publication.

